# Enhancing public health response: a framework for topics and sentiment analysis of COVID-19 in the UK using Twitter and the embedded topic model

**DOI:** 10.3389/fpubh.2024.1105383

**Published:** 2024-02-21

**Authors:** Aisha Aldosery, Robert Carruthers, Karandeep Kay, Christian Cave, Paul Reynolds, Patty Kostkova

**Affiliations:** ^1^Centre for Digital Public Health in Emergencies, Institute for Risk and Disaster Reduction, University College London, London, United Kingdom; ^2^Department of Computer Science, University College London, London, United Kingdom

**Keywords:** COVID-19, sentiment analysis, Twitter, public response, United Kingdom, topic modeling, embedded topic model, government policy

## Abstract

**Introduction:**

To protect citizens during the COVID-19 pandemic unprecedented public health restrictions were imposed on everyday life in the UK and around the world. In emergencies like COVID-19, it is crucial for policymakers to be able to gauge the public response and sentiment to such measures in almost real-time and establish best practices for the use of social media for emergency response.

**Methods:**

In this study, we explored Twitter as a data source for assessing public reaction to the pandemic. We conducted an analysis of sentiment by topic using 25 million UK tweets, collected from 26th May 2020 to 8th March 2021. We combined an innovative combination of sentiment analysis via a recurrent neural network and topic clustering through an embedded topic model.

**Results:**

The results demonstrated interpretable per-topic sentiment signals across time and geography in the UK that could be tied to specific public health and policy events during the pandemic. Unique to this investigation is the juxtaposition of derived sentiment trends against behavioral surveys conducted by the UK Office for National Statistics, providing a robust gauge of the public mood concurrent with policy announcements.

**Discussion:**

While much of the existing research focused on specific questions or new techniques, we developed a comprehensive framework for the assessment of public response by policymakers for COVID-19 and generalizable for future emergencies. The emergent methodology not only elucidates the public’s stance on COVID-19 policies but also establishes a generalizable framework for public policymakers to monitor and assess the buy-in and acceptance of their policies almost in real-time. Further, the proposed approach is generalizable as a tool for policymakers and could be applied to further subjects of political and public interest.

## 1 Introduction

COVID-19 was first identified in 2019 and declared as a worldwide emergency in January 2020 (1). To mitigate the spread of the virus, governments around the world put several public health restrictions in place. In particular, the UK government enacted three separate periods of national lockdown beginning March 2020, November 2020, and January 2021 (2) alongside a range of policies, such as social distancing and the use of mask (3). Assessing the public’s response to, and compliance with, these policies is crucial. To this end, surveys have been conducted by the UK’s Office for National Statistics (ONS) (4). However, these surveys can be costly in terms of time and resources.

We explored the potential of Twitter as a data source for this information. Social media and Twitter in particular, have emerged as a significant source of data regarding the public’s response to the pandemic (5–7). Twitter’s advantages include a high volume of relevant data and the potential for flexibility and low cost in a successful approach. Previous research has identified correlations (8) between Google search trends and COVID-19 incidence. Although Google searches are pertinent, they primarily indicate information needs or search, and lack crucial elements, such as sentiment toward government policy. Our approach allowed us to precisely pinpoint sub-topics and trends within a broader topic, and to accurately associate sentiment with to those topics – an analysis not feasible with Google search trends alone.

Earlier studies have examined COVID-19 content on Twitter via Natural Language Processing (NLP) methods (9, 10). However, existing research on COVID-19-related Twitter content has limitations such as relatively brief study periods (11–13), expensive annotation methods (14), and a lack of clarity and interpretability in topics (15). We anticipated that Twitter data would reveal sufficient sentiment for us to assess the public’s overall response to certain events and thus trained a model to analyze sentiment on our behalf. A sentiment model could be trained on a small selection of manually labeled tweets and then quickly deployed on the entire tweet corpus. Since we were interested not only in overall sentiment, but also in topic-specific sentiment, we first grouped the data into topics before applying the sentiment classifier. We adopted the Embedded Topic Model (ETM) (16), noted for its ability to uncover interpretable topics even amidst extensive vocabularies that include rare words and stopwords.

**Figure 1:**
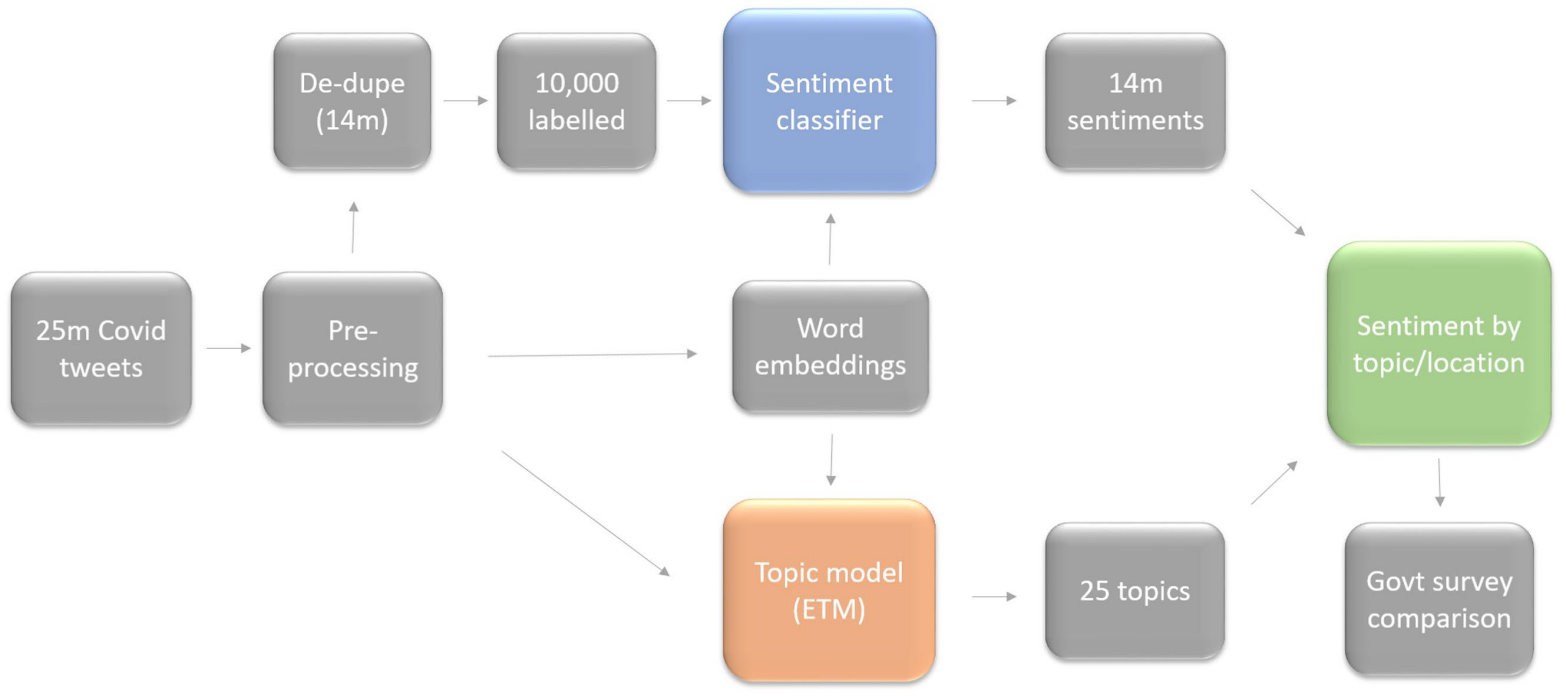
Schematic diagram of the proposed approach.

This fusion of a supervised sentiment model with an unsupervised topic model allowed us to understand the impact of policy changes and announcements on different aspects of public life, as well as to make comparisons with national surveys reflecting real-world behavior. Figure 1 depicts the overall process. Our approach distinguished by its highly customized application of techniques contrasted with standard approaches in this field, its extended study duration, and it’s clear and interpretable results. Our study possesses a distinctive combination of characteristics that enhance data analytics in the following ways:

- Advanced and novel combination of NLP techniques, including custom word embedding, improved topic modeling using ETM over LDA, neural networks for sentiment classification rather than VADER, and probabilistic approach to topic-sentiment assignment for weighted signals.
- Tracking data over an extended period (9 months – 25 million UK tweets).
- Dataset labeling guided by best-practice standards.
- A variety of results correlated with news and announcements, including location-based results, as well as compared to government behavioral surveys.
- A generalizable and flexible framework as opposed to an excessively narrow experimental design.

Furthermore, to the best of our knowledge, no previous study has presented exhaustive an approach that correlates analytical findings with public health policy to aid policymakers. This has yielded the following principal contributions:

- We obtained signals from Twitter within distinct topic clusters pertaining to significant real-world pandemic events.
- We evidenced that the Embedded Topic Model yielded more distinct topics from our dataset than legacy methods.
- We established a link between Twitter insights and health policy in an unprecedented manner, employing official ONS surveys as benchmarks to shed light on the public’s reaction to policies during the COVID-19 pandemic as well as offering a methodology for policymakers to evaluate public engagement and approval of their policies virtually almost in real-time.
- We offer a comprehensive methodology that addresses problems with previous studies using a combination of NLP techniques, encompassing the diverse topics associated with the central theme, in a reproducible framework that is scalable and generalizable to future scenarios (i.e., pandemics or other public policy responses).

This investigation, while centered on COVID-19, demonstrates the versatility of the methodology for other crises where timely assessment of public sentiment of government policies is crucial, This framework provides an indispensable instrument for policy-makers dealing with future emergencies.

The remainder of this manuscript is organized into five main sections. Section 2 presents a critical review of the related work, contextualizing our study within the current body of literature. Section 3 describes the methodology employed, detailing data collection, processing, and analysis techniques. In Section 4, we discuss the results, interpreting the significance of sentiment signals and their correlation with policy events. Section 5 explores the broader implications of our findings for public health policy and sentiment analysis while acknowledging the limitations of our study. Finally, Section 6 concludes the paper, by summarizing key contributions and suggesting directions for future research.

## 2 Related work

### 2.1 Public opinion about COVID-19 on twitter

Since the start of the pandemic, Twitter has been recognized as an important source of data and large datasets have been collected (17–20). Sentiment has been studied over the time period for specific topics such as vaccines (21), but this approach lacks generality, as it is limited to predefined research topics, rather than allowing the major topics of discussion to emerge from the data in an unsupervised manner. Several studies have attempted to track emotional responses to the pandemic (10, 11, 22–25). However, a major methodological problem with this is that emotions are not well understood scientifically, with even so-called “basic” emotions not having been shown to exist in consistent terms across languages, cultures and subjective experiences (26). This calls for a simpler and more reliable method to measure human reactions. Furthermore, another limitation of the emotion-based approach is that it needs to be combined with specific topics over time to isolate exactly what people are reacting to.

The complexity of a crisis such as the COVID-19 pandemic means that simply measuring overall sentiment is likely to miss the sentiment signal around particular topics and thus be too blunt an instrument for policymakers. Although several attempts have been made at Covid topic modeling on Twitter (9, 15, 27, 28), there has generally been a lack of clarity and distinctiveness in the topics produced. The authors of (8) mention interpretability problems and compute limitations for their graphical methods. It can be seen that their topics lack distinction, and they claim that a manual hyperparameter search could help with this. The authors of (14) express doubt about their topic results, saying that “the scientific quality of the themes should be further validated.” Indeed, their best approach produced only six topics (albeit over a short time period), and “China,” “Chinese” or “Wuhan” appear in the top ten words of five out of those six topics. The authors of (25) similarly concede that “the interpretation of topics is a challenging task.” That paper pre-clustered by sentiment, and then found separate topics within each group, thus giving separate positive, neutral and negative topics. This approach is flawed because it does not leverage the whole dataset for topic analysis. The topic word clouds presented the paper are visibly noisy and lack the clarity that we were able to achieve. In terms of measuring the response to government policy, a Dutch study showed a change in sentiment over time toward mask-wearing (29), but did not perform topic modeling, instead simply using the existence of the word “mask” as a filter. This is unsatisfactory because it did not leverage the potential of topic modeling to cluster related tweets. Also, their method relied on annotators answering a specific question comparing the tweet content to the policy of a Dutch health organization. This is a highly cumbersome procedure which does not generalize well due to the necessity for bespoke experiment design and annotator training. A UK study examined sentiment toward masks (30), but did not compare it to official government surveys on behavior. A North American study (31) relied on the involvement of public health experts for its aspect-based sentiment approach. It is undesirable for a method to rely on experts for implementation, because they may not be available, or their cost may be prohibitive.

In contrast, our approach does not rely on a high degree of specific domain knowledge for implementation (30). ANTi-Vax (32) presented a Twitter dataset and model to identify vaccine misinformation. However, not only did it also rely on medical experts for its construction, it focused on a single challenge only (vaccine misinformation), rather than the wider spectrum of COVID-19 topics. Our work attempts to address these limitations in a cohesive manner. We measured public opinion simply in terms of positive, negative, or neutral sentiment, which is less subjective and more generalizable than emotion, and investigated the dataset using a combination of Topic Modeling and Sentiment Classification. Our approach covered many topics, ranking and analyzing them to provide a comprehensive study of public responses on Twitter.

### 2.2 Topic modeling

Topic modeling on large corpora is often carried out using Latent Dirichlet Allocation (LDA) a generative model which learns a distribution over topics together with a distribution over the vocabulary for each topic (33). Indeed, LDA has been the predominant method used for topic modeling on COVID-19 Twitter datasets (12, 13, 24, 28, 30, 31). Many extensions to LDA have been proposed (14) for topic modeling, but existing studies have not explored these methods despite their relevance (14, 34). During the pandemic, many normal, everyday words took on new meanings. An example of this is the word “bubble,” which was used to denote the social circle you were allowed to keep during the lockdown periods. Noting the unique nature of our dataset, we took care to retain as much information as possible. Consequently, pre-compiled stopwords were not removed, and instead a specific corpus of stopwords were removed by looking at the most and least frequent occurring words in the corpus as suggested by (35), which found that removing conventional stopwords (“and,” “the,” etc.) was detrimental to a sentiment classifier for Twitter data. Therefore, unlike LDA, we required methods which were impervious to stop words. We used the Embedded Topic Model (ETM) (16), as it claimed to discover interpretable topics even with large vocabularies that include rare words and stopwords.

The ETM discovers topics in embedding spaces by modeling words as categorical distributions, setting the parameter of each distribution as in the inner product of the word embedding and the learned embedding of a given topic. Similar to LDA, the ETM learns a probability distribution over k topics, with each topic represented by a probability distribution over the vocabulary of the entire dataset. This can be used to compute the probability that a tweet was generated by each of the k topics, creating a soft, probabilistic assignment of a tweet to each topic. Due to their similarity, LDA was suitable to be used as a performance benchmark for the ETM. Our results demonstrated the superiority of the ETM model over LDA, which has been traditionally employed in this task. Both the ETM and our sentiment classifier take embedded representations of tweets as input, allowing for some consistency over our approach.

### 2.3 Sentiment classification

Twitter Sentiment Analysis (TSA) has been noted as being particularly difficult compared to normal text and has historically not performed well (36). Tweets are short, previously limited to 140 characters and with a UK limit of 280 characters during our data collection period. This restriction encourages the use of compact expressions, hashtags, and emojis. It is possible for the user to output several related tweets in a row, but due to the limitations of the Twitter API, related tweets are unlikely to be captured. Twitter is also characterized by sentiment class imbalance, with a large majority of neutral tweets (which was indeed the case in our dataset). Spam and commercial messages are common (37). The way sentiment is expressed may vary considerably by topic, and the COVID-19 pandemic brought its own terminology. The meta-study in (36) emphasized data preprocessing of items such as emoji, hyperlinks, and hashtags. Stopwords differ on Twitter and their treatment affects performance (38). Many COVID-19 Twitter studies have used Valence Aware Dictionary for sentiment Reasoning (VADER) (39) to perform sentiment analysis (12, 13, 24, 40, 41). However, it should be noted that VADER is a generic method that relies on a pre-defined dictionary and a set of valence rules. Given the nature of our dataset, which contained many Covid-specific terms, VADER has the drawback of potential inaccuracy in terms of identifying tweet-sentiment relationships in our context (30). In contrast, we trained the neural network model on a set of annotated Covid tweets, using a custom word embedding. This allowed for accurate identification of sentiment in the context of the pandemic (30, 42).

### 2.4 Labeling tweets

In the domain of Twitter sentiment analysis, a crucial facet of social media analytics, several tools and techniques has been utilized for data labeling. Advanced Natural Language Processing (NLP) instruments, such as the Bidirectional Encoder Representations from Transformers (BERT), offer sophisticated algorithms for text analysis, capable of understanding complex language patterns and sentiments (43–45). In parallel, TextBlob offers a more accessible sentiment analysis by producing a polarity score that ranges from-1 to 1, thus allocating emotions into neutral, negative, and positive categories [Ref]. Other approaches may classify emotions into more specific categories like anger, joy, fear, and sadness, providing a finer granularity of the emotional spectrum expressed in social media discourse (44, 45).

Crowdsourcing platforms, such as Amazon Mechanical Turk, represent an alternative approach, where labeling tasks are distributed among human annotators. This human-in-the-loop approach can be particularly advantageous for tasks requiring nuanced understanding of language, irony, or cultural contexts that automated systems may not fully grasp. However, while such tasks benefit from the diverse interpretations of a broad workforce, this can also lead to a lack of consistency, with disparate annotators potentially interpreting identical instructions differently. Thus, MTurk can be a powerful tool for human labeling, but like any method, it has its trade-offs and may not be suitable for all projects (43, 46, 47).

NLP tools offer scalability and efficiency; however, there are instances, such as during emergent events like the COVID-19 pandemic or when analyzing newly coined terminologies, where manual labeling by humans can provide superior accuracy, particularly when working with tweets subject to 280-character constraints. Each method carries its own set of benefits and limitations, and the choice between automated or manual labeling often depends on the specific requirements of the research, the nature of the data, and the desired level of precision in sentiment analysis. Thus, both automated tools and human judgment play pivotal roles in the evolving landscape of sentiment analysis, with researchers often selecting a hybrid approach to leverage the strengths of both methodologies (48). Best practices for labeling thus include using multiple annotators assessing the same tweet and, either keeping only tweets with full agreement or weighting the annotators based on a measure of their skill compared to a gold standard (49).

As a result, we opted for manual labelling of our tweets dataset, ensuring quality control and allowing for a rapid response, This decision was driven by the need to conduct the labeling in early days of the emergency when new public health policy terminology was daily emerging, thus, humans would outperform pre-trained models. However, the work presented in this study is a novel a framework where specific components, such as the labeling methodology, could be modified to fit a different situation as/if appropriate.

## 3 Methodology

### 3.1 Data collection and preprocessing

Twitter provides an API that enables data scraping. This API offer access to tweets, which are text strings of up to 280 characters published to the network, along with relevant metadata. For our study, we utilized the Twitter Stream API to collect a corpus of tweets spanning from May 26th, 2020 to March 8th 2021. We specifically collected tweets that included one or more of the 60 English keywords relevant to the pandemic, a comprehensive compilation of keywords in various languages, which were utilized for the purpose of collecting tweets, can be found in the provided footnote.^1^ Although the ideal approach to identifying UK tweets was through geo-tagging, such instance was rare. UK tweets were therefore filtered by the English language (auto-detected by Twitter) and a pre-specified list of UK user locations (a free-text optional field). We expect that this will capture the majority of UK tweets in the dataset, except where the user location field is blank. The entire filtered UK dataset contained 25.1 m Tweets. This number includes repeated retweets; when these were removed the number dropped to 14.6 m unique tweets. We estimated that this sample represented <1%^2^ of all relevant UK tweets in that period, due to the limitations of the API and lack of geo-tagging. This is a conservative estimate of UK tweet Locations were standardized into the UK Nomenclature of Territorial Units for Statistics regions, using a combination of manual mappings and inverted indices on the free-text location. Approximately 15% of dates in the data range were missing due to technical issues. The Tweet object encompasses a comprehensive set of fundamental attributes at the root level, including id, created_at, text, etc. The principal attributes derived from the tweet encompass the following:

- text: denoting the substantive textual content of the tweet itself,
- created_at: indicating the precise timestamp of tweet creation,
- id: serving as a unique identifier for the tweet,
- geo: provide geolocation data, if available,
- lang: signifying the abbreviated form denoting the language employed in the tweet,
- user: encapsulating the comprehensive profile details of the tweet’s author,
- favorite_count and retweet_count: represents the total number of favoriting and retweeting of the tweet.
- entities: encompassing diverse elements such as URLs, @-mentions, hashtags, and symbols.

Tweet text was tokenized and preprocessed to identify the most common emoji and to remove most punctuation, hyperlinks, and non-alphabetic characters. Lemmatizing, stemming and n-gram construction was not done. Instead, we used a bespoke word embedding learned from the dataset in the Topic Modeling process. Where relevant, we removed unknown words that occurred only once. The omission of lemmatizing, stemming, and n-gram construction in sentiment analysis and topic modeling using Twitter datasets is primarily attributed to the challenges and potential ambiguities associated with these techniques. Twitter data often contains various forms of noise, such as misspellings, abbreviations, slang, and emoticons. These factors make it challenging for lemmatizing or stemming techniques to effectively handle the noise, potentially resulting in the grouping of unrelated words or the generation of incorrect word forms. Furthermore, the informal and conversational language style prevalent on Twitter makes these techniques less suitable for accurately capturing the meaning or sentiment expressed in informal tweets. Additionally, the removal or alteration of emoticons and emojis, which serve as important sentiment indicators on Twitter, can lead to the loss of valuable information. It is worth noting that n-gram construction, although capable of capturing contextual and sequential information in text, may encounter issues of sparsity and overfitting when applied to short and noisy Twitter messages (50).

### 3.2 Labeling tweets

A good quality training dataset is essential for successful Twitter Sentiment Analysis. As the research was undertaken at the beginning of global COVID-19 emergency when new public health policy terms were daily emerging thus manual labeling was deemed more accurate ty, as in these situations humans outperform automated pre-trained models, and provide results rapidly. As the size of our training dataset was smaller due to limits on the team (in real-word situations, conducted by WHO or Departments of Health, this will not be an issue) the result was further enhanced by word embedding, described in the next section.

Four of the authors manually labeled 10,000 tweets, uniformly sampled from the start to the end of the period, with each team member labeling 2,500 tweets. Following an initial test run where the same set of tweets was labeled by all four authors, we collaborated to set labeling rules to mitigate individual differences that had been identified between the authors.

Thus, tweets were labeled to be either Negative (−1), Neutral (0) or Positive (1). A tweet had to be clearly and unambiguously negative or positive to avoid being labeled as neutral. Tweets that appeared to be ironic or sarcastic were labeled as a human should interpret them. Annotators were not allowed to assume any missing words, and any retweets were to be judged as if they were original tweets. There was also a review procedure for borderline cases. The resulting proportions were: Negative 18.1%, Neutral 74.3%, Positive 7.6%.

### 3.3 Skipgram embeddings

As outlined above, throughout the COVID-19 pandemic, many words have taken on new meaning in order to describe novel situations, for example, “bubble.” Commonly used pre-trained word embeddings, such as those trained on news articles (51) or tweets before the pandemic (52), were unlikely to have adequately captured the re-purposing of existing words in combination with the use of Twitter-specific language. To address this, we used the gensim library (53) to create 300-dimesional Word2Vec embeddings using the skipgram architecture, with 10 epochs, a minimum frequency of 50, negative sampling of 10 and a window size of 4. Words occurring less often in the corpus than the minimum frequency were removed before training. Noting that our unique dataset contained a rich vocabulary of rarely used words, the value of the minimum frequency was set much higher than the default values (∼ 2). The resultant embeddings displayed pleasing qualitative results, with many words taking on Covid-specific meaning, with other words retaining existing meaning (Table 1). However, many shortened versions of words appeared as an artifact of the truncation of tweets received from the API. These embeddings can be used with both downstream tasks, topic, and sentiment modeling. Through a qualitative review, we felt that this retained the niche information in the dataset and would enhance the performance of both tasks.

**Table 1:** Embedded word similarities.

Word	Three most similar
Coronavirus	covid, coronav, coronavi
ppe	ppe?, ppe!, equipment
social	distancing, socia, soc
soup	lentil, tomato, pickled

### 3.4 Topic modeling

There were a number of considerations in training the Embedded Topic Model (ETM). Firstly, whether to train word embeddings during the topic modeling process, the standard Unlabeled ETM, or use pre-computed word embeddings, known as Labeled ETM (16). Secondly, the number of topics the model should discover and finally, the minimum and maximum document frequency, that is, the minimum number of tweets a word must appear in, and the maximum number of tweets a word can appear in, to be used in the model. By using model perplexity, a measure of how well a probability distribution predicts a sample, as a metric, optimal model type and topic number could be investigated empirically by finding the model which scored the lowest perplexity. Following this, the quality of a resulting model could be investigated further using quantitative and qualitative methods.

Qualitatively, the embeddings used/trained during the ETM process were assessed by considering the closest words to keywords, such as “mask,” “covid” and “ppe,” in the embedding space, and whether these most similar words captured the similarities we might have expected. Quantitatively, we followed the approach of (16), defining a Topic Quality metric as the product of Topic Diversity, the percentage of unique words in the top 25 words across all topics, and Topic Coherence, the average pointwise mutual information between the top 10 words in each topic. A model which scored the lowest perplexity, highest Topic Quality and which used qualitatively acceptable embeddings could then be deemed as the best performing model.

### 3.5 Sentiment classifier

The 10, 000 labeled tweets were split uniformly randomly into a training set of size 8,000 and validation and test sets each of size 1,000, with proportionately distributed labels. We used a recurrent neural network (RNN) structure for the sentiment model. Tweets were mapped through the bespoke 300-dimensional embedding layer, followed by a 32-dim gated recurrent unit (GRU) layer with ReLU activation and 3-dim linear layer with SoftMax activation. The embedding layer was created as part of the Topic Modeling and was not further trained. Tweets were randomized and sorted by length before training in batches of 10. We found that our results deteriorated if tweets were not sorted by length, due to the necessity to pad shorter tweets with Unknown tokens. Due to the class imbalance, we used a weighted variant of the cross-entropy loss. The loss attributed to the tweet was weighted inversely proportionally to the relative frequency of its label. The training took place over 200 epochs. In this experiment, we used the Adam optimizer (54) with a learning rate of 0.001 which is set to the Pytorch library (55), it was observed that varying the learning rate did not have a significant impact on the performance of the model.

## 4 Model development and experiments

### 4.1 Topic model development

Due to a desire to retain as much information as possible, and noting that ETM is robust to stopwords, the minimum document frequency was set to be as small as possible given a 25GB constraint on RAM usage. This value was found to be 10,000. Models were trained for 50 epochs, at which point perplexity was no longer decreasing materially. We trained for both the standard Unlabeled and Labeled-ETM models, for a range of topic numbers and learning rates for optimization. Notably, in the case of the unlabeled ETM, the most optimal configuration was achieved with 7 topics. Topic diversity, coherence and quality are summarized in Table 2 for the best performing models. From a range of topics numbers we trained the models for, the best perplexity minimizing and quality maximizing model was found to be Labeled-ETM with 25 topics, trained with a learning rate of 0.001. With a topic quality of 0.067, our final model outperformed all other models, including LDA and the unlabeled ETM equivalent. Interestingly, LDA scored a higher topic diversity. Overall, in the presence of stopwords, the ETM was able to find topics of higher coherence out of the models run, reproducing results found by (16).

**Table 2:** Topic quality for the top three models.

Model (Topics)	Coherence	Diversity	Quality
Label. ETM (25)	0.086	0.781	0.067
Unlabel. ETM (7)	0.066	0.834	0.054
LDA (25)	0.004	0.854	0.003

### 4.2 Sentiment classifier development

During the development of the sentiment model, we tested several variations. First, we used word embeddings trained on a larger corpus of UK Twitter data over the period 2012–2016 (dimension 512) (52). However, we found that many of the most important words from our dataset were missing, simply because many of these words were created later than the training period for the embeddings. Furthermore, the meaning and usage of many existing words has changed due to the COVID-19 pandemic, and these new relationships could not be captured by the embeddings. The creation of our own custom word embeddings (dimension 300) alleviated both of these problems and improved model performance. Table 3 shows the classification distribution across the dataset using models trained on either of these two embeddings.

**Table 3:** Sentiment classification distribution using two different UK Twitter word embeddings.

Label	Embedding	
	Generic	Bespoke
Negative	16.4%	18.1%
Neutral	77.1%	73.6%
Positive	6.5%	8.3%

The apparent similarity masked qualitative differences in the embeddings.

At the surface level there is high agreement, but further analysis showed that the models agreed only 78.6% of the time. The bespoke embedding model performed slightly better on the validation dataset, and qualitative review of tweets showed that it did indeed identify sentiment better in some Covid-related tweets. However, this was at the cost of misclassification on others, and it may have been more appropriate to label a given tweet as neutral if both models did agree. We did not pursue this further due to our limited validation data. We trained the model on both removing and not removing stopwords, the removal of which has been shown to reduce the ability of the model to identify negative sentiments (35).

In our case, the difference in prediction accuracy was very low, probably due to the small size of the training dataset. We considered different architectures by changing the number of hidden neurons, GRU layers, and linear layers. We also looked at other common recurrent types: vanilla RNN and an LSTM. The combination we settled upon gave the best validation performance (as measured by the metrics shown in Table 4), although many networks of higher dimensions gave similar results. We had a preference for the simplest model for performance reasons that was optimal perhaps due to the limited size of our training dataset. We also considered the degrees of regularization via dropout and different mini-batch sizes.

**Table 4:** Metrics for sentiment model.

		Actual label			Macro avg.
		Negative	Neutral	Positive	
Training data (8,000 tweets)	Precision	87.5%	96.8%	84.9%	89.7%
	Recall	90.5%	95.8%	86.2%	90.8%
	F1	89.0%	96.3%	85.6%	90.3%
Validation data (1,000 tweets)	Precision	46.7%	82.5%	40.2%	56.5%
	Recall	50.3%	80.4%	42.1%	57.6%
	F1	48.4%	81.5%	41.1%	57.0%
Test data (1,000 tweets)	Precision	44.9%	84.5%	37.9%	55.8%
	Recall	51.2%	79.9%	47.8%	59.6%
	F1	47.8%	82.1%	42.3%	57.4%
Proportions		18.1%	74.3%	7.6%	

## 5 Results

### 5.1 Sentiment classification

To evaluate the performance of the sentiment classifier, we computed the average precision, recall, and F1 scores across three datasets, namely Training, Validation, and Testing, results displayed in Table 4. The validation data indicates that the model was only somewhat reliable at identifying sentiment. Qualitative review of results was quite promising; the model seemed to do a reasonable job of classification despite a low amount of training data.

The classification ability of the model was likely enhanced by the use of the word embedding trained on the full dataset. Qualitative review of individual tweets found that positively classified tweets tended to contain positive and inclusive language, while those negatively classified tended to contain aggressive, emotive and persuasive language, and political terms. Certain terms seemed to affect model accuracy, including terms associated with the National Health Service (NHS) in the UK and political terms. An example was “overwhelmed,” which appears to cause incorrect negative classification, probably due to its association with the NHS. The model sometimes did not treat “not” as an adverb correctly. The model was also worse at picking up negative sentiments that did not use strong aggressive and emotive language. Even though model results may have varied at an individual tweet level, over a large sample we expected sentiment signals to come through. This was the basis of our time trend analysis, described in the following section.

### 5.2 Sentiment by topic over time

**Figure 2:**
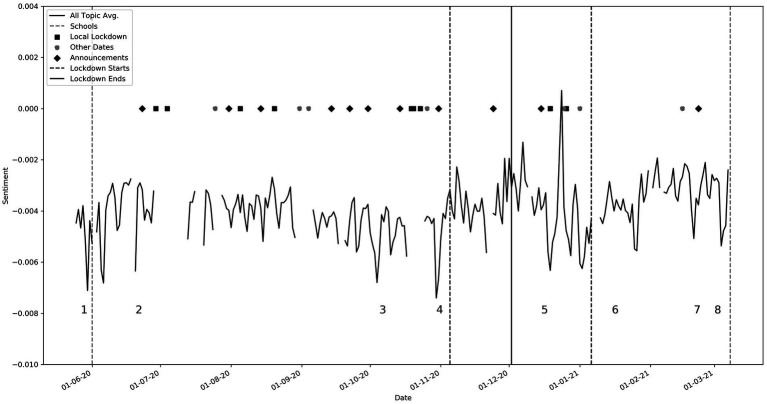
Average sentiment time series, with key events labeled as: (1, 2) reaction to George Floyd’s death (Topic 18), (3) Increase in COVID-19 cases (04/10/2020, Topic 8), (4) Second lockdown announcement (11/11/2020, Topics 1, 6, 19, 20, and 25), (5) Series of December policy announcements (Topics 1, 6, 19, 20, and 25), Christmas Day (Topic 21), (6) Third lockdown announcement (06/01/2021, Topics 1, 6, 19, 20, and 25), (7) Court Hancock ruling (25/01/2021, Topics 1, 6, 7), (8) Budget and NHS pay rise (03/03/2021, Topic 7). Please refer to Table 5 for topic keywords.

Timeliness and relevance of specific timely events are essential for social media real-time analysis. Having labeled each tweet with a sentiment label, we were able to map probabilistic interpretations of the topics to the words in that tweet. We were then able to gauge average sentiment by topic over the dataset. This weighted approach contrasted with previous approaches that assigned tweets to only one topic (24, 30), or separated topics by overall sentiment (28), both of which greatly oversimplified the richness of the data. The weighted approach allowed for a more nuanced and accurate picture of topic-based sentiment over time, because the sentiment expressed in a particular tweet could contribute to multiple topics. Figure 2 corresponds to average sentiment over time with points of interest labeled numerically.

**Figure 3:**
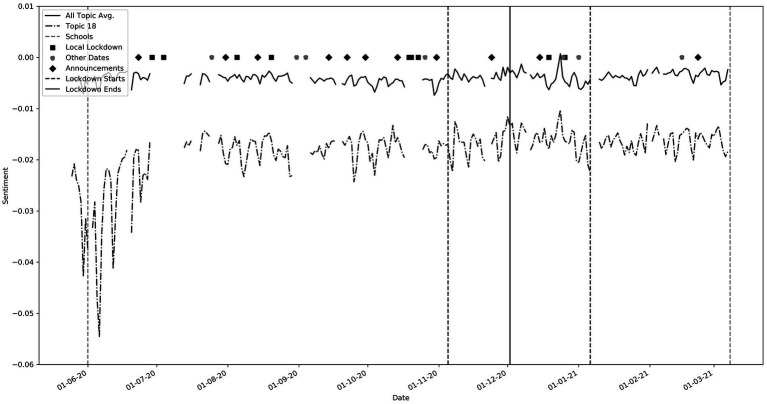
Topic 18 (Keywords: ‘people’, ‘every’, ‘police’, ‘black’, ‘single’, ‘women’, ‘lives’, ‘human’, ‘right’, ‘dead’). Note the large trough around 25th May 2020, corresponding to the death of George Floyd.

Specific events were pre-chosen as points of interest. These include government announcements, local lockdowns, lockdown periods, non-COVID-19 events, and school openings. The discontinuous nature of the graph is due to missing data. Associations can clearly be found between the topics and key events. The main peaks and troughs are listed beneath Figure 2 with the relevant supporting topics, the keywords for which can be found in Table 5. In summary, points 1 and 2 correlate to the death of George Floyd, matching troughs in topic 18 (see also Figure 3). This was a non-covid related event, but the negative sentiment was clearly so great that it penetrated the overall dataset. Point 3 correlates to when COVID-19 cases jumped up by 23,000 on one day due to catching up with a backlog of cases. Points 4 and 5 relate to an increase in negative sentiment during key government announcements.

**Table 5:** Top ten words, symbols, and emoji (in bold) by topic.

Topic	Words
1	‘news’,‘covid’,‘uk’,‘coronavirus’, ‘scotland’, ‘bbc’, government’,‘media’,‘rules’,‘bbcnews’
2	‘last’,‘year’,‘years’,‘week’,‘days’,‘months’,‘two’,‘weeks’,‘ago’,‘today’
3	‘well’,‘ive’,‘got’,‘never’,‘ever’,‘done’,‘seen’,‘back’,‘best’,‘weve’
4	‘would’,‘think’,‘get’,‘like’,‘make’,‘much’,‘time’,‘really’,‘say’,‘going’
5	‘trump’,‘us’,‘state’,‘party’,‘president’,‘house’,‘labour’,‘said’,‘vote’,‘realdonaldtrump’
6	‘government’,‘johnson’,‘boris’,‘ppe’,‘tory’,‘deal’,‘public’,‘brexit’,‘tories’,‘hancock’
7	‘nhs’,‘staff’,‘working’,‘workers’,‘pay’,‘work’,‘doctors’,‘thank’,‘lives’,‘save’
8	‘people’,‘covid’,‘virus’,‘many’,‘died’,‘spread’,‘risk’,‘flu’,‘young’,‘corona’
9	‘support’,‘school’,‘amp’,‘schools’,‘open’,‘children’,‘free’,‘help’,‘business’,‘students’
10	‘amp’,‘world’,‘borisjohnson’,‘country’,‘us’,‘government’,‘britain’,‘history’,‘brexit’,‘around’
11	‘covid’,‘test’,‘amp’,‘positive’,‘trace’,‘testing’,‘patients’,‘tests’,‘hospital’,‘nhs’
12	‘pandemic’,‘covid’,‘crisis’,‘global’,‘due’,‘many’,‘people’,‘hit’,‘middle’,‘us’
13	‘would’,‘think’,‘get’,‘make’,‘like’,‘much’,‘time’,‘thats’,‘going’,‘really’
14	‘dont’,‘know’,‘get’,‘cant’,‘anyone’,‘need’,‘think’,‘believe’,‘let’,‘tell’
15	‘covid’,‘deaths’,‘uk’,‘cases’,‘new’,‘coronavirus’,‘death’,‘rate’,‘number’,‘infection’
16	‘amp’,‘family’,‘friends’,‘story’,‘video’,‘twitter’,‘watch’,‘talk’,‘show’,‘long’
17	ellipsis,‘like’,tearsofjoy,rofl, ‘oh’,‘fuck’,‘fucking’,‘shit’,cryingface,‘look’
18	‘people’,‘every’,‘police’,‘black’,‘single’,‘women’,‘lives’,‘human’,‘right’,‘dead’
19	‘covid’,‘coronavirus’,‘uk’,‘new’,‘second’,‘lockdown’,‘response’,‘wave’,‘countries’,‘governments’
20	‘vaccine’,‘covid’,‘health’,‘first’,‘vaccination’,‘public’,‘uk’,‘vaccines’,‘says’,‘pfizer’
21	‘day’,‘good’,‘morning’,‘christmas’,‘today’,‘time’,‘great’,‘hope’,‘see’,‘happy’
22	‘covid’,‘new’,‘read’,‘important’,‘report’,‘data’,‘latest’,‘impact’,‘study’,‘article’
23	‘mask’,‘wear’,‘wearing’,‘people’,‘masks’,‘keep’,‘social’,‘go’,‘youre’,‘home’
24	‘please’,‘amp’,‘us’,pointdown,‘looking’,handclap,‘follow’,‘share’,‘rt’,‘join’
25	‘lockdown’,‘th’,‘pm’,‘london’,‘restrictions’,‘st’,‘march’,‘place’,‘city’,‘new’

**Figure 4:**
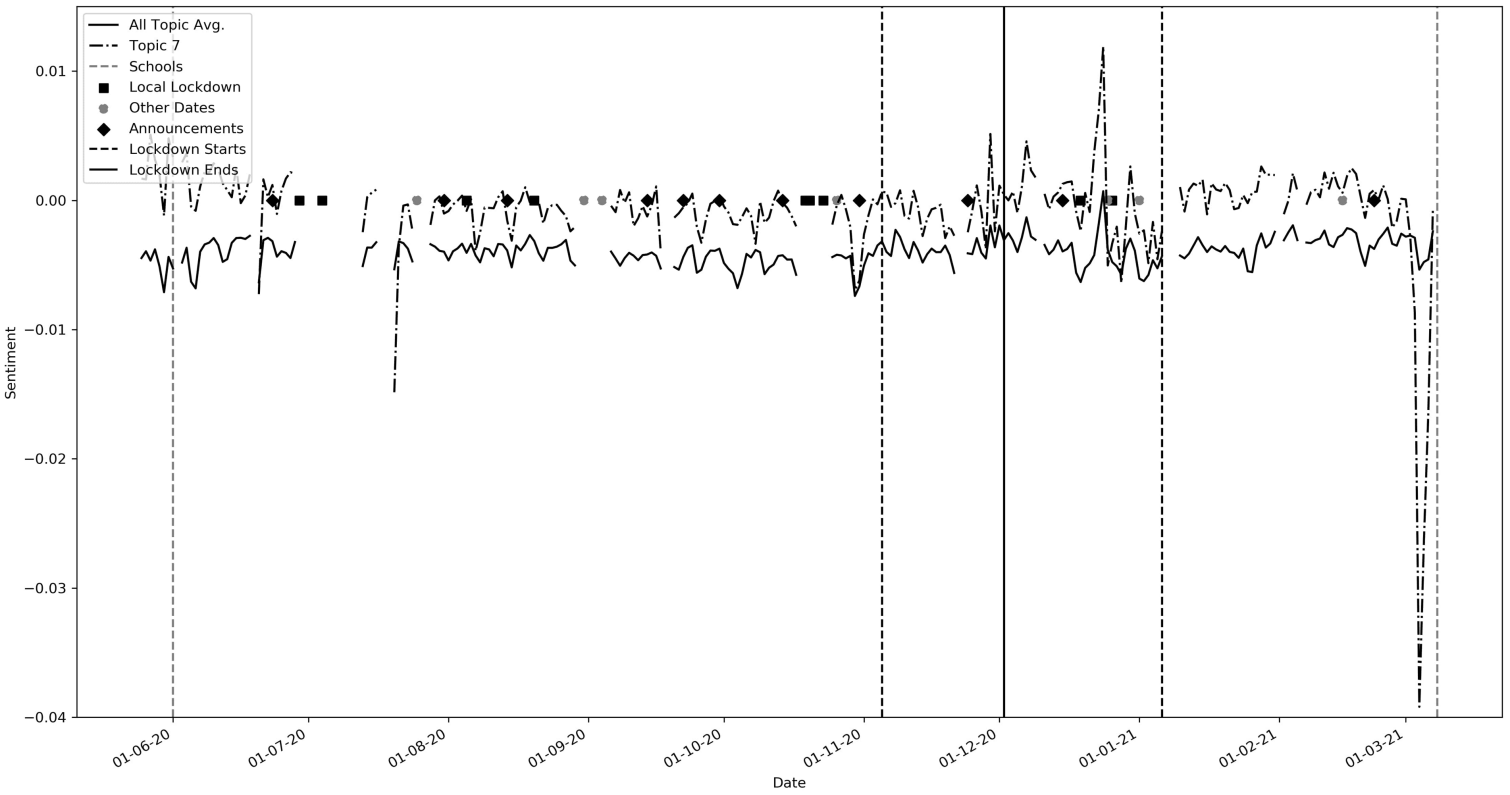
Topic 7 (Keywords: ‘nhs’, ‘staff’, ‘working’, ‘workers’, ‘pay’, ‘work’, ‘doctors’, ‘thank’, ‘lives’, ‘save’). Note the large trough on 3rd March 2021 during the spring budget speech announcing a low NHS pay rise.

While these troughs can be seen in most of the topics, they are most pronounced in topics that have “lockdown” or “government” keywords such as topics 1, 6, 19, 20 and 25. Throughout this period, sentiment for topics 1, 6 and 19 (not shown) was well below the average sentiment. These contain words pertaining to “government,” “johnson,” “hancock” and “news.” Topic 20, with keywords such as “vaccine,” “pfizer” and topic 25 which has words such as “lockdown,” “london” and “restrictions” were more positive than the general trend. Interestingly, a spike in negative sentiment occurred just before the announcement of lockdowns at points 4 and 5. This could suggest social media speculation before the actual announcements. We observe that average sentiment after the third lockdown began (Point 6) was higher than the average sentiment in 2020. This perhaps reflects increased optimism regarding the pandemic. Point 7 correlates to events relating to Matt Hancock, the Health secretary, and again corresponding pronounced troughs could be seen in topics that include “government” and “hancock” (not shown). Point 8 correlates to the spring budget date when a pay rise of only 1% was given to the NHS. A very large trough is seen in topic 7 which contains “NHS” (See also Figure 4). We found many other interesting relationships not included here, including reactions to free school meals, Donald Trump and vaccines.

### 5.3 Comparison to UK governmental compliance surveys

**Figure 5:**
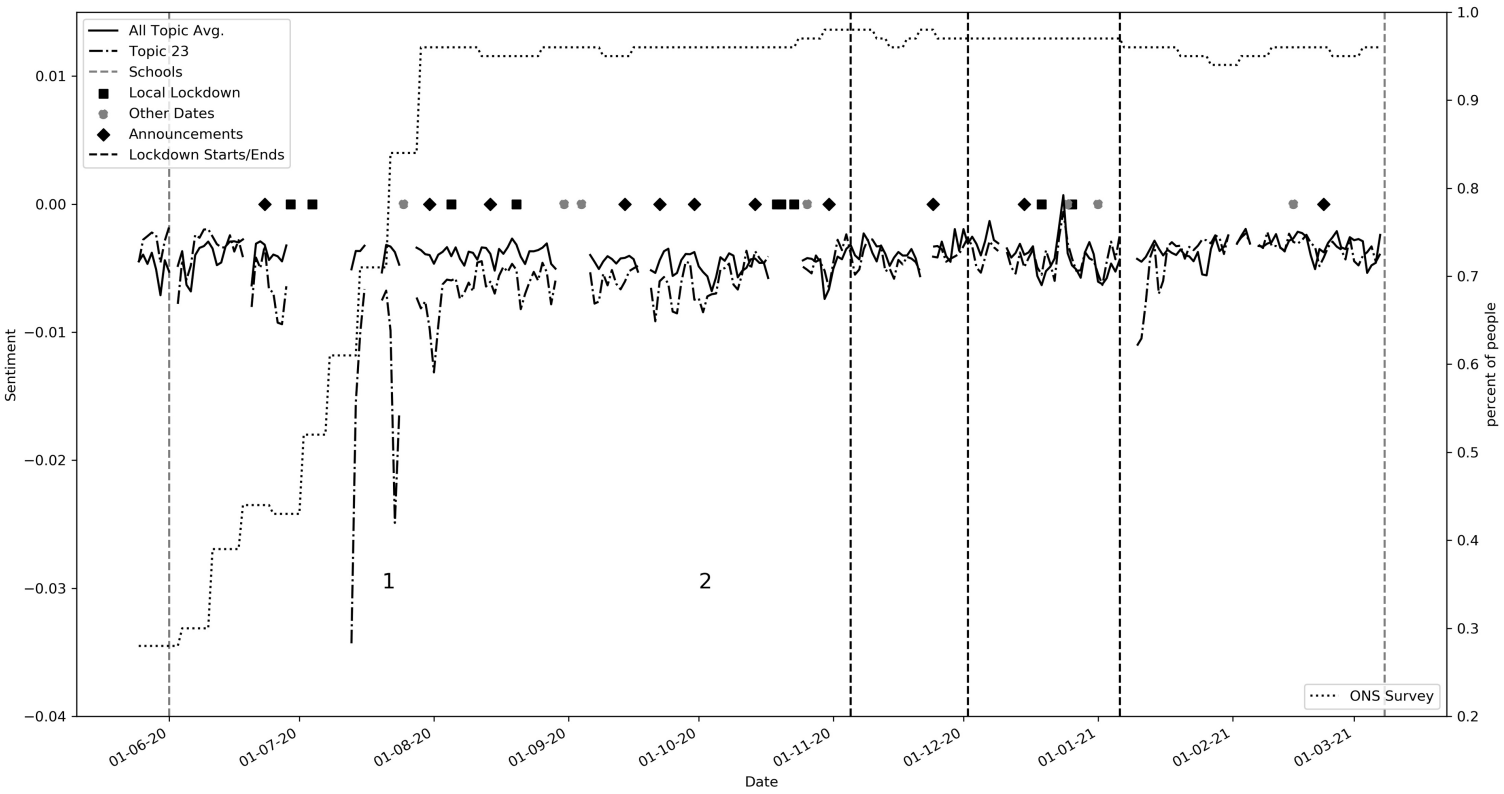
Topic 23 compared to ONS survey: Percentage of adults that have used a face covering when outside their home in the past seven days (Keywords: ‘mask’, ‘wear’, ‘wearing’, ‘people’, ‘masks’, ‘keep’, ‘social’). The keys labeled in the graph showed (1) the large spike in negative sentiments when masks became mandatory, and (2) the sentiment gradually becomes in line with the average sentiment.

The UK Office for National Statistics (ONS)^3^ carried out compliance surveys throughout the pandemic. This provides a high-quality, objective information source about real-world behavior (4). In Figure 5, we compare the sentiment for topic 23 which contains words such as “mask,” “wear,” “wearing,” to the ONS survey metric “Percentage of adults that have used a face covering when outside their home in the past seven days.” The dotted line relates to the ONS survey and shows that the percentage of adults complying slowly climbs up until the end of July when compliance remains high. During this period, we can see that the average sentiment for this topic is lower than the overall average. At (1) denoted in the graph, we see a sharp decline in sentiment. On 14th July 2020 it became mandatory to wear masks in shops and on transportation. While some data is missing, we see that after this period the sentiment slowly starts to become more in line with the average sentiment by (2). This perhaps suggests an initial backlash, followed by acceptance. This is supported by consistently high compliance scores during this period.

### 5.4 Sentiment by topic and location

We also analyzed the sentiment by the user-defined location in the user’s Twitter profile as commonly used (37) as geo-tagged tweets were a very small minority. Thus, this technique would not be suitable for our study. Overall, the average sentiment shape was similar by region. However, there were some notable differences. An example was Topic 1 (Keywords include “news,” “covid,” “uk,” “coronavirus,” “scotland”). On the 11^th^ of September 2020 Nicola Sturgeon announced that tougher restrictions would need to be in place across Scotland after a period of local lockdowns. This announcement relates solely to Scotland, and we isolated a significant drop in sentiment to users self-defined as being in Scotland. In comparison, London did not have the same reaction.

## 6 Discussion

### 6.1 Summary of findings

Our approach combined topic modeling and sentiment analysis over time and was effective in detecting responses to real-world events. Key differentiators in our approach included the use of a labeled COVID-19 UK tweet dataset, the Embedded Topic Model, and a neural network sentiment classification model (rather than the standard approaches of LDA and VADER, respectively), a custom word embedding, and probabilistic topic-sentiment assignment for weighted signals. The novel combination use of the Embedded Topic Model and bespoke word embedding allowed for the unique features and language of our dataset, leading to interpretable signals. In comparison, prior work has had difficulty obtaining interpretable topics (9), connecting sentiment to specific external causes and time periods (11), or comparing to real-world behavior without highly specific investigations (30). Along with the relative clarity of the topics found, the strengths of our approach include its flexibility and generality, allowing it to be applied to further use cases. Topic-sentiment-time-geographic relationships provide a rich resource for comparison to mainstream news and government surveys and may have the potential to contribute to future measurement of the tweeting public’s opinion.

### 6.2 Strengths of our approach

In our study, we acknowledge the potential presence of overfitting in our model, as evidenced by its superior performance in training data compared to the validation and test data. However, several important factors need to be considered. Firstly, our study distinguishes itself by employing a rigorous labeling process that involves multiple annotators and strict rules to establish the ‘true’ tweet labels. This process enables clear identification of the gold standard human performance, which is unsurprisingly unattainable given the relatively small size of our dataset. Additionally, most studies in the field do not provide comparable metrics or strive to reach such standards. Secondly, the majority of existing studies rely on the VADER model for sentiment classification, which is based on rule-based techniques and lacks training on true sentiment labels as we have done. Therefore, our study adheres to higher standards in terms of accuracy and overfitting considerations. Moreover, Twitter Sentiment Analysis is a well-known challenging task, and our study’s promising results occur despite the limitations imposed by the dataset size. We contend that the achieved results reflect the effectiveness of our methodology.

While acknowledging that results may vary for individual tweets, our primary focus lies in capturing the overall average sentiment across millions of tweets. In contrast to classification tasks such as spam filtering, where precise performance is crucial due to less ambiguous categories, our study places greater emphasis on the broader sentiment trends exhibited by a large-scale dataset. An important and subtle feature of our methodology is the incorporation of a custom word embedding, trained on a vast dataset of over 10 million tweets. While human sentiment labels were only available for 8,000 tweets, the generalization capacity of our model is significantly enhanced by leveraging word similarity from the word embedding when applied to the full dataset. We posit that the impressive results obtained in our study can be attributed, in part, to this generalization ability, enabling the model to maximize the use of a relatively low training sample. The combined effect is key to this study. The results are acceptable, noting that, the model successfully identifies roughly half of the minority positive/negative tweets, and 80% of the majority neutral ones. Lastly, we have conducted a comprehensive range of experiments, including variations in model architecture and regularization techniques, ultimately opting for a relatively simpler architecture due to the lack of substantial performance gains from more complex models. It should be noted that the main objective of our paper is to establish a framework applicable to multiple domains, allowing for further adaptations and advancements of the model.

In light of the aforementioned points, it becomes evident that the raw sentiment classifier results should be interpreted within the broader framework of our methodology. While these results may initially seem limiting, they provide valuable evidence supporting the effectiveness of our approach, considering the constrained nature of our labeled data. It is worth highlighting that our overall methodology capitalizes on the utilization of a custom word embedding trained unsupervised on the entire dataset, enhancing the generalization capabilities of our model. Furthermore, it is important to note that the majority of referenced studies in our literature review do not provide comparable performance metrics, further emphasizing the novelty and rigor of our evaluation approach. By considering these factors collectively, we can confidently assert the robustness and significance of our findings.

### 6.3 Implications for public health

Over a decade, social media have been used instrumental for studying human response and sentiment to public health events (7), public health communication (56) and misinformation (57). This study introduced a versatile framework designed to support policymakers in the field of public health. The generalizability of the framework enables application to a range of emergency scenarios, and public policy domains where decisions affect large parts of the society or the whole country(ies). Pandemics are the most typical examples but similar approach would be needed in case of a biological attack, toxic substance leak, or a dramatic shift in policy on driving, parking, smoking or vaccination. The real-time insights into public sentiment, enables policymakers to rapidly gauge policy buy-in and acceptance levels. By capturing nuanced signals from social media, it provides a deep understanding of the evolving public response to health policies. In summary, this framework serves as a powerful tool for public health policymakers when rapid, and adaptable sentiment insights are needed in real-time.

### 6.4 Limitations

The quality and rigor of the proposed framework is largely defined by our limitations, which are in our case, data volume and topic content.

#### 6.4.1 Volume

The manual labeling of data was not only time-consuming but also necessitated a robust process to ensure both consistency and accuracy. While this approach was adopted to construct a labeled dataset for training and evaluation, it is crucial to recognize that manual labeling introduces subjectivity and demands substantial resources, especially in rapidly evolving contexts like the COVID-19 pandemic. An area of potential improvement lies in expanding our training and validation sets, allowing larger network architectures and better more effective hyperparameters fine-tuning. Still, it is essential to acknowledge that our dataset exhibited inherent noise, and it is uncertain whether an increase in dataset size would have yielded significant enhancements in model performance.

#### 6.4.2 Topic content

Being the result of an unsupervised algorithm, the topics found do not have definitive interpretations. In addition, topics may “drift” over time. The topic model used data from the entire time period. For future use, it would be necessary to continually collect sufficient relevant data as the situation changed, and to adapt the method for online use by using dynamic time windows (34). Despite the limitations of the work, the proposed approach could also be applied to other subjects of political, and public interest using different keywords that are relevant to the subject.

### 6.5 Ethical considerations

#### 6.5.1 Annotation

Although we manually annotated tweets for sentiment, it may have been possible to replace or augment this with a pre-trained classifier. It should be noted that concerns have been raised about the use of platforms such as Amazon Mechanical Turk, including low wages and lack of proper licensing and consent mechanisms (46).

#### 6.5.2 Representation

Analysis of the British Social Attitudes Survey 2015 (58) found that UK Twitter users are more likely on average to be male, under 30 and from managerial, administrative, and professional occupations. Large sectors of British society are likely to be underrepresented.

#### 6.5.3 Applications

Our approach is designed to infer public opinions and views from the aggregation of millions of tweets. There is potential for a government, or indeed, Twitter itself, to use such an approach as part of a population surveillance system. This could be used to target groups or individuals who spread content deemed to be unacceptable, with either positive or negative consequences. Although individual tweets are public, the invisible nature of such surveillance could have implications for privacy, democracy, or human rights, and prevent the open use of Twitter.

The authors assert that all procedures contributing to this work comply with ethical standards, and the University College London Ethics Committee (code: 4147/002) approved all procedures.

### 6.6 Future work

Next, direction of this research would include further refinement of the topic modeling and different methods could be investigated to benchmark our findings. The topic-sentiment reaction of the tweeting public to new government policy announcements could be investigated further, as the technique is generalizable to any public policy irrespective of domain and demonstrates results in almost real-time. The strength of the methodology is its generalizability – this method provides a blueprint not just for an application to Covid pandemic but for real-time assessment of public response and sentiment for new policies. We have seen indications of a relationship between some events and sentiment stability (divisiveness). It would be interesting to study this, although we expect that finer temporal sampling than daily might be required. The method could also be applied to other time periods using different keywords, where different topics would be expected. Finally, expanding the training dataset is considered significant for improving the model’s performance. The current study acknowledges the relatively small size of the labeled dataset used for training. Increasing the dataset size can provide the model with a more diverse and representative set of examples, enabling it to learn more effectively and generalize better to unseen data.

## 7 Conclusion

This study developed a novel framework for assessing citizens’ reaction to public health policies from social media discourse during the COVID-19 emergency to inform policymakers about the sentiment and buy-in. Our objective of this novel study was to determine whether clear signals could be obtained from Twitter that illustrated public opinion during the pandemic and response to various real-world events by combining Twitter analytics with a data collected by the ONS survey. We analyzed topic sentiment across 25 topics and various UK regions by integrating the output from a recurrent network and a topic clustering model. A clear and interpretable per-topic sentiment signal was observed. Due to the richness of the topic model, we could directly correlate specific peaks and troughs in sentiment with events and announcements. The combined model has demonstrated different responses by user defined locations to events that impact those locations. In addition, we found a relationship between sentiment in the model and real-world compliance with wearing face coverings, according to an ONS survey. Our study shows how an integrated approach, coupled with attention to detail at each stage of the process, from tweet labeling to refinement of machine learning techniques, can result in distinct separation of signal from the diverse and noisy tweets that characterize a global pandemic. Our work introduces a novel approach, combining topic modeling and sentiment analysis by leveraging the Embedded Topic Model and a neural network sentiment classification model, allowing for greater generalization and stronger results. Future work will aim at enhancing the model’s performance through regularization techniques and expanding the training dataset to further improve accuracy and reliability. The methodology proved robust to indicate citizens’ responses to public health policies in almost real-time and could be generalizable to any other major public policy events.

## Data availability statement

The raw data supporting the conclusions of this article will be made available by the authors, without undue reservation.

## Ethics statement

The studies involving humans were approved by University College London Ethics Committee (approval number 4147/002). The studies were conducted in accordance with the local legislation and institutional requirements. Written informed consent for participation was not required from the participants or the participants’ legal guardians/next of kin in accordance with the national legislation and institutional requirements.

## Author contributions

KK, AA, and PK: conception and design of the work and final approval of the version to be published. AA: data collection. KK, RC, CC and PR: design and development of the topic and sentiment analysis methods, tweet labelling, data analysis, interpretation of findings and drafting the article. AA and PK: critical revision of the article. All authors contributed to the article and approved the submitted version.

## Footnotes

^1^https://zenodo.org/record/8018264

^2^https://www.internetlivestats.com/twitter-statistics/, https://www.statista.com/statistics/242606/number-of-active-twitter-users-in-selected-countries/

^3^https://www.ons.gov.uk/
